# Development of an abdominal wall abscess caused by fish bone ingestion: a case report

**DOI:** 10.1186/s13256-019-2301-7

**Published:** 2019-12-15

**Authors:** Kiyomitsu Kuwahara, Yasuji Mokuno, Hideo Matsubara, Hirokazu Kaneko, Mikihiro Shamoto, Shinsuke Iyomasa

**Affiliations:** 1 Department of Surgery, Yachiyo Hospital, 2-2-7, Sumiyoshi-cho, Anjo-shi, Aichi 446-8510 Japan; 2 Department of Pathology, Yachiyo Hospital, 2-2-7, Sumiyoshi-cho, Anjo-shi, Aichi 446-8510 Japan

**Keywords:** Fish bone, Abdominal wall abscess, Foreign body ingestion, Laparoscopic surgery

## Abstract

**Background:**

A small percentage of patients with foreign body ingestion develop complications, which have a variety of clinical presentations. Less than 1% of cases require surgical intervention. We present a patient with an abdominal wall abscess resulting from a fish bone that pierced the cecum. The patient was treated laparoscopically.

**Case presentation:**

A 55-year-old Japanese man presented to our hospital with a complaint of right lower abdominal pain. A physical examination revealed tenderness, swelling, and redness at the right iliac fossa. Computed tomography showed a low-density area with rim enhancement in his right internal oblique muscle and a hyperdense 20 mm-long pointed object in the wall of the adjacent cecum. Based on the findings we suspected an abdominal wall abscess resulting from a migrating ingested fish bone. He was administered antibiotics as conservative treatment, and the abscess was not seen on subsequent computed tomography.

Two months after the initial treatment, he presented with the same symptoms, and a computed tomography scan showed the foreign body in the same location as before with the same low-density area. We diagnosed the low-density area as recurrence of the abdominal wall abscess. He underwent laparoscopic surgery to remove the foreign body. His appendix, and part of his cecum and the parietal peritoneum that included the foreign body, were resected. He had an uneventful postoperative course, and at 1 year after the surgery, the abdominal wall abscess had not recurred.

**Conclusions:**

An abdominal wall abscess developed in association with the migration of an ingested fish bone. We suggest that a laparoscopic surgical resection of the portion of the bowel that includes the foreign body is a useful option for selected cases.

## Background

Foreign body ingestion sometimes occurs accidentally and is usually related to food, such as fish and chicken bones, and includes ingestion of toothpicks. Most foreign bodies pass uneventfully through the gastrointestinal tract within a week. In a small percentage of cases, a foreign body impacts, penetrates, or perforates the gastrointestinal tract [1]. Endoscopic intervention is required in 10 to 20% of patients, and surgical intervention to remove a foreign body is required in less than 1% of patients [2]. Here we report the case of a patient who was treated laparoscopically for a pyogenic abdominal wall abscess that resulted from migration of an ingested fish bone into the wall of the cecum. Therefore, we should be aware that an abdominal wall abscess can develop in association with an ingested foreign body, such as a fish bone. Moreover, our laparoscopic approach for resecting a portion of bowel containing a foreign body is a useful option for selected cases.

## Case presentation

**Fig. 1:**
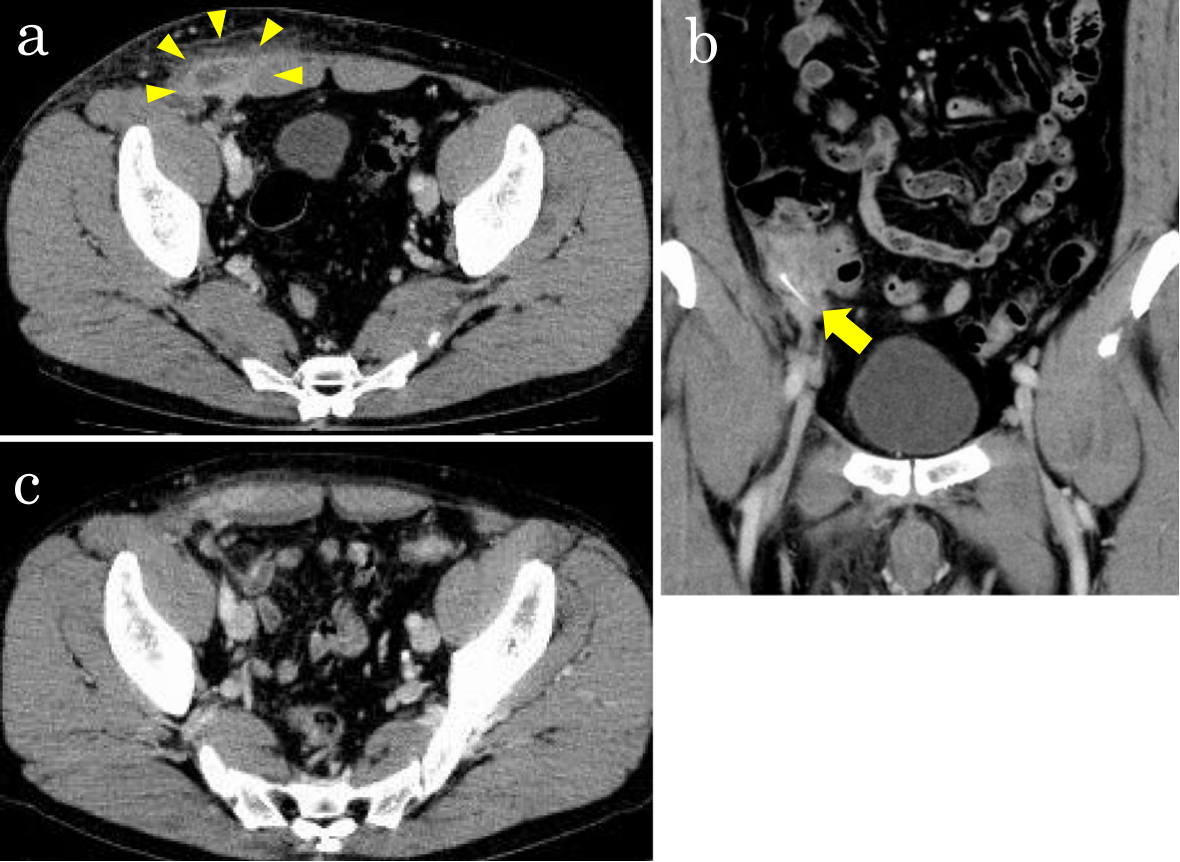
**a** Computed tomography scan shows a low-density area with rim enhancement in the right abdominal wall (arrowheads), and **b** a hyperdense, sharp object in the cecal wall adjacent to the low-density area (arrow). **c** After 2 weeks of antibiotic administration, the abscess is no longer apparent

A 55-year-old Japanese man presented to our hospital with a complaint of right lower abdominal pain. His medical and family histories were unremarkable. He worked in a factory. He occasionally consumed alcohol and smoked cigarettes. He denied having eaten fish during the previous few days. Three days prior to visiting the hospital, he noticed redness of the skin and pain involving his right lower abdomen. A physical examination revealed tenderness, swelling, and redness at the right iliac fossa; however, he was afebrile (36.5 °C). His blood pressure and pulse were 122/80 mmHg and 85 beats per minute (bpm), respectively. A laboratory examination revealed an increased white blood cell (WBC) count of 10.4 × 10^3^ cells/μL and C-reactive protein (CRP) level of 10.19 mg/dL. Except for this finding, laboratory testing revealed no abnormal values. Computed tomography (CT) showed a 42 × 22 mm low-density area with rim enhancement in his right internal oblique muscle (Fig. 1a), and a 20 mm-long hyperdense, sharply pointed object in the wall of his cecum adjacent to the low-density area (Fig. 1b). Although he was unaware of having ingested a sharply pointed object such as a fish bone, we suspected that the object was a fish bone because of the shape. Thus, the findings were diagnosed as abdominal wall abscess due to a foreign body piercing the cecum. The abscess was aspirated, but did not return fluid. A blood culture had no growth.

He was treated conservatively with flomoxef (2 g/day) for 2 weeks. After the treatment, his WBC and CRP level returned to normal, and the abdominal wall abscess was not seen on CT (Fig. 1c). His symptoms of tenderness, swelling, and redness at the right iliac fossa also improved. The hyperdense pointed object remained in the same location. Therefore, 24 days after the diagnosis, he underwent colonoscopy for removal of the object; however, no abnormality was visualized in his cecum. Although surgical removal was indicated, we monitored our patient carefully without performing surgery, based on a mutual agreement with our patient.

**Fig. 2:**
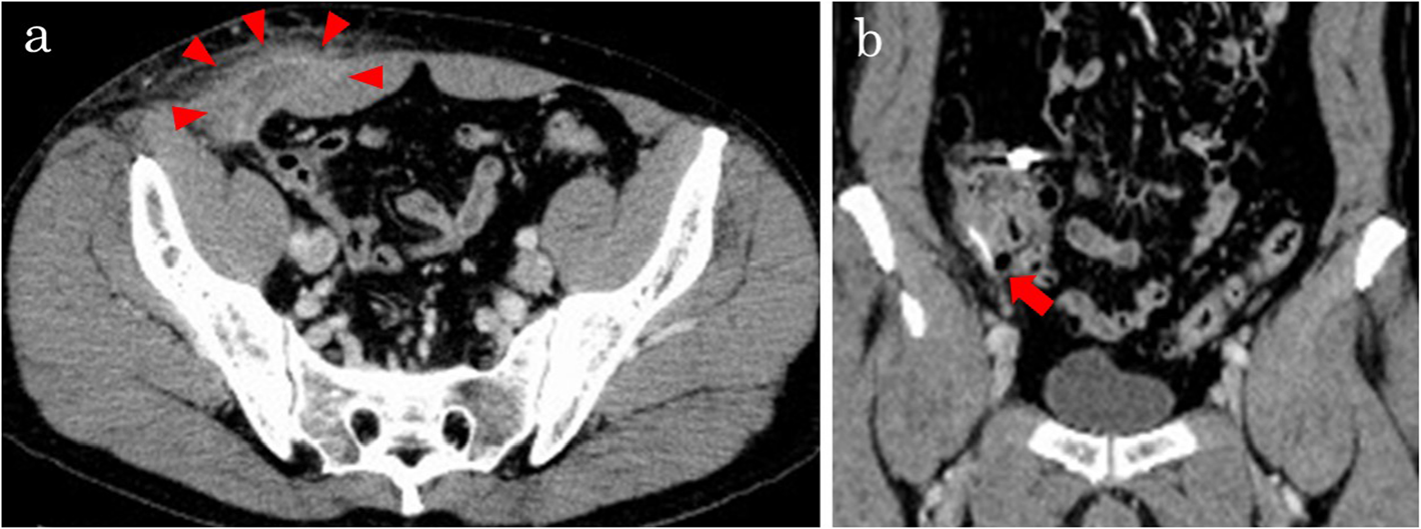
Two months after the initial treatment. **a** Computed tomography scan shows the same low-density area in the same location (arrowheads). **b** The foreign body remains in the same location (arrow)

Two months after the initial treatment, he presented again with right lower abdominal pain. CT showed the same low-density area and the foreign body in the same location in the cecum (Fig. 2a, b); the findings were diagnosed as recurrence of the abdominal wall abscess due to the foreign body. Laparoscopic surgery was performed to remove the object. He was treated with flomoxef (2 g/day) for 7 days before laparoscopic surgery, and a subsequent laboratory examination revealed that his CRP level had decreased from 8.57 to 0.82 mg/dL. When he presented immediately before undergoing surgery, he no longer complained of right lower abdominal pain.

**Fig. 3:**
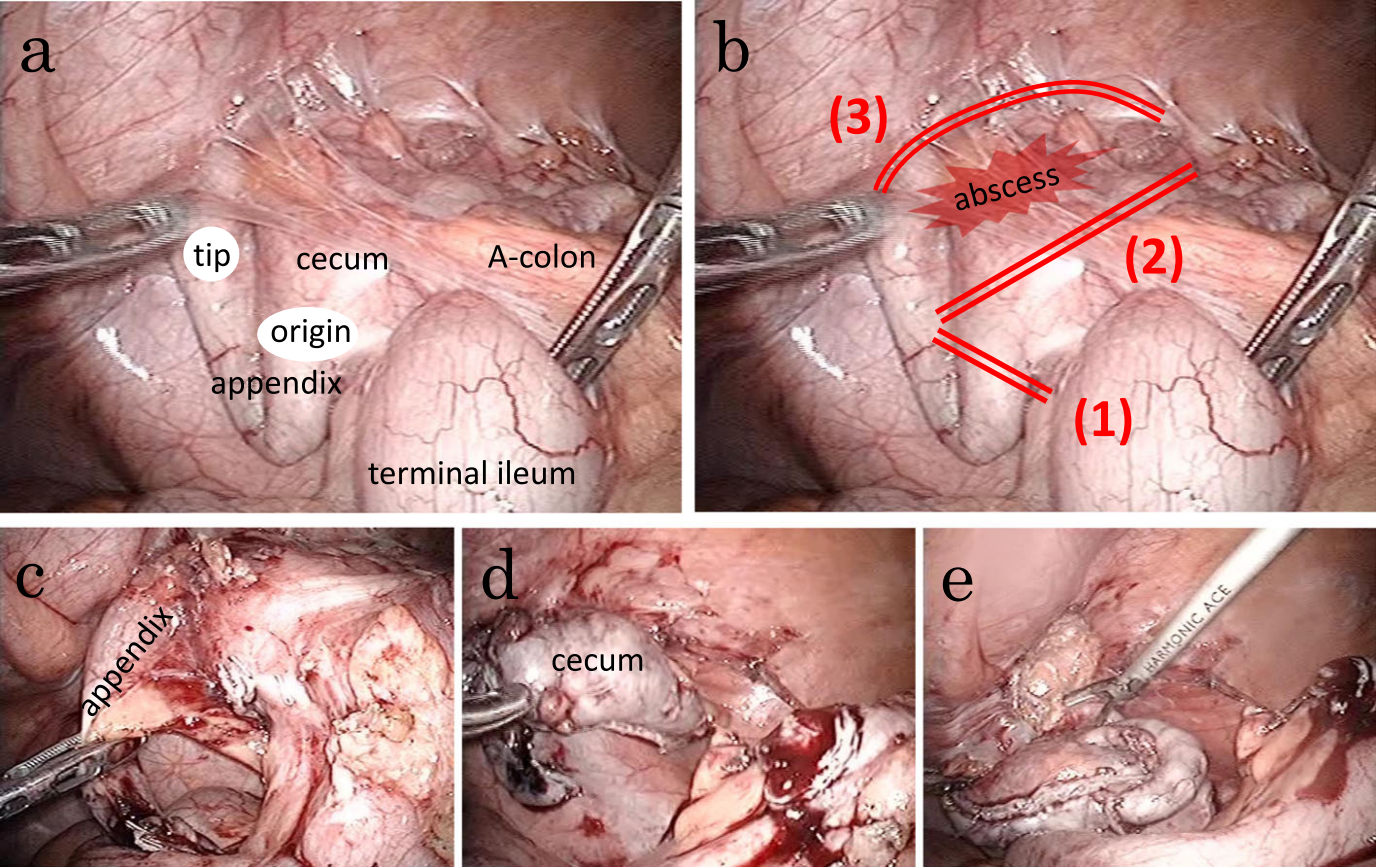
**a** Laparoscopic image of fibrous adhesions between the cecum, tip of the appendix, and the right parietal peritoneum. **b** Planned resection lines appear as double lines (*1*)–(*3*). **c** The appendix was dissected at its origin (*line 1* in **b**). **d** Adherent cecum was dissected at *line 2* in **b**. **e** En bloc resection was performed at *line 3* in **b**

**Fig. 4:**
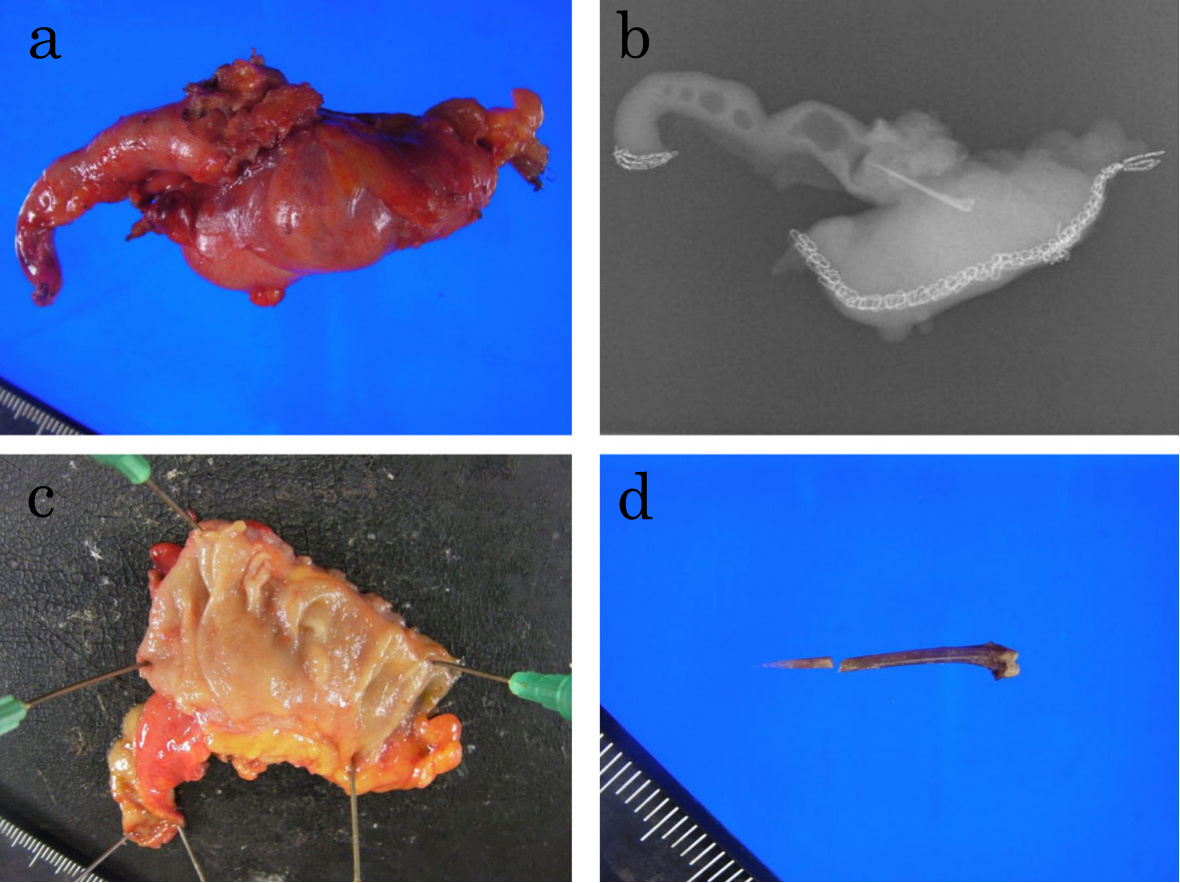
**a**, **b** The specimen and X-ray findings. The sharp object was located between the appendix and the cecum. **c** No mucosal lesion is seen in the resected specimen, neither in the cecum nor the appendix. **d** The object was identified as a 2 cm-long fish bone

The laparoscopic findings included fibrous adhesions between the cecum, tip of the appendix, and right parietal peritoneum (Fig. 3a). Our resection lines were based on the preoperative CT and the laparoscopic findings (Fig. 3b). First, we dissected the appendix at its origin using iDrive™ (Medtronic Minneapolis, Minnesota, USA) (Fig. 3c), then we dissected the adherent section of the cecum using iDrive® (Fig. 3d), and then performed an en bloc resection of the foreign body with parietal peritoneum (Fig. 3e). Finally, we confirmed removal of the foreign body by a plain X-ray examination of the resected specimen (Fig. 4a, b). We then placed a percutaneous drain in the abscess. A mucosal lesion was seen neither in the cecum nor the appendix of the resected specimen (Fig. 4c) and most of the object was located in the wall of the cecum, except for the pointed tip, which had penetrated the tip of the appendix. The object was identified as a 2 cm-long fish bone (Fig. 4d). These findings suggest that the fish bone pierced the cecal wall and then migrated within the cecal wall. After surgery, our patient was treated with flomoxef (2 g/day) for 4 days, and he had an uneventful postoperative course and was discharged on postoperative day 10. One year after surgery, he is doing well, without recurrence of the abdominal wall abscess.

## Discussion

Foreign body ingestion is usually related to food [3], with fish bones frequently being ingested accidentally, especially among populations that frequently eat fish [4]. Ingested fish bones may be forgotten, and there can be a time lag of months or even years between ingestion and the onset of symptoms. In previous reported cases of abscess formation due to fish bone ingestion, most patients denied any history of fish bone ingestion. Therefore, a clinical history might not be helpful [5, 6]. Moreover, fish bone complications in the gastrointestinal tract manifest with a variety of clinical presentations, ranging from impaction in the upper gastrointestinal tract, dysphagia, bowel obstruction, and silent perforation to frank peritonitis [7]. Peritonitis can be acute or chronic. However, only a few cases of abdominal wall abscess due to fish bone ingestion have been reported [8–10]. Therefore, the medical history and clinical presentation alone do not provide information suggestive of fish bone ingestion.

By contrast, with recent advances in the image quality of CT, the ability to identify a fish bone in a lesion has improved [11]. In most of the reported cases, CT revealed not only inflammatory lesions but also linear hyperdense objects in the lesions [12–14]. In our case, our patient was unaware of ingesting a fish bone. However, CT revealed the fish bone-like object adjacent to the abdominal wall abscess. Thus, in patients with presentations suggesting the presence of an inflammatory lesion of unknown cause, we should not exclude the possibility of fish bone ingestion, even if the patient does not report a history of fish bone ingestion.

The management of an ingested foreign body depends on the patient’s signs and symptoms, and the type and location of the ingested object. In almost all asymptomatic patients, conservative management is appropriate, since most objects will pass uneventfully [15]. In all cases of a foreign body in the esophagus, the foreign bodies require removal within 24 hours, even without the presence of signs or symptoms, because the risk of complications increases dramatically with time [16]. Sharp objects in the esophagus should be removed urgently, because of the high risk of perforation. Even if sharp objects pass through the stomach, complications have been described in up to 35% of patients [17]. Moreover, objects in the stomach larger than 2 to 2.5 cm in diameter or longer than 5 to 6 cm should be removed, because they will not pass through the pylorus, duodenum, or ileocecal valve. The objects should be removed endoscopically if possible. Surgical removal should be considered in patients who develop complications, and for a foreign body that does not progress through the digestive tract [18].

In our patient, careful observation after conservative therapy with antibiotics seemed to be an acceptable treatment option for an asymptomatic ingested foreign body that was not identified during colonoscopy. However, the abdominal wall abscess was recurrent, and the foreign body did not progress through the digestive tract. Based on CT imaging, we decided that laparoscopic removal of the foreign body was safe and possible, and we subsequently performed laparoscopic surgery to remove the foreign body.

There are several reported cases of abdominal abscesses caused by a foreign body in various regions, including the liver, pancreas, gastrointestinal tract, and abdominal cavity [19–23]. Laparoscopic removal was performed in some cases [22, 23]. Even in those cases, however, the foreign body was removed by blunt dissection under exploratory laparoscopy. Moreover, a few cases of abdominal wall abscess due to foreign body perforation have been reported [8–10]. In those cases, the foreign bodies were located in the abscess, thus, curettage of the abscess and removal of the foreign body were performed via laparotomy. For our patient, we planned the resection based on the preoperative CT images, and performed laparoscopic surgery. As described previously, we removed the foreign body with part of the cecum, the appendix, and the parietal peritoneum for the following reasons: the preoperative CT scans revealed that almost all of the foreign body was in the wall of the cecum. Removal of the foreign body by blunt dissection only might have led to damage that would have been difficult to repair via a laparoscopic approach. Furthermore, an appendectomy and partial resection of the cecum never leads to stenosis of the gastrointestinal tract. To the best of our knowledge, no previous cases have been published that describe a laparoscopic resection of a foreign body that had caused an abdominal wall abscess. Moreover, the uncomplicated postoperative course of our patient supports the feasibility of our laparoscopic resection.

## Conclusions

In conclusion, we describe the first laparoscopically treated case of a pyogenic abdominal wall abscess resulting from a fish bone migrating into the wall of the cecum. Even if a patient is unaware of ingesting a foreign body, we should be aware that an abdominal wall abscess can develop in association with an ingested fish bone that has migrated to the region and then remained adjacent to the abscess. Moreover, our case suggests that our laparoscopic approach for performing a resection of the portion of the bowel containing a foreign body, which was planned based on the results of diagnostic imaging, is a useful option for selected cases. Such cases include those such as ours, with a foreign body that can be identified by CT.

## Data Availability

Not applicable.

## Abbreviations

CRP: C-reactive protein
CT: Computed tomography
WBC: White blood cell
